# Combination of laser microdissection, 2D-DIGE and MALDI-TOF MS to identify protein biomarkers to predict colorectal cancer spread

**DOI:** 10.1186/s12014-019-9223-7

**Published:** 2019-01-22

**Authors:** Chandra Kirana, Lifeng Peng, Rose Miller, John P. Keating, Corinne Glenn, Hongjun Shi, T. William Jordan, Guy J. Maddern, Richard S. Stubbs

**Affiliations:** 1 Discipline of Surgery, The Queen Elizabeth Hospital, Basil Hetzel Research Institute, University of Adelaide, 37a Woodville Road, Woodville, SA 5011 Australia; 2 Wakefield Biomedical Research Unit, Wakefield Clinic, Wakefield Hospital, Wellington, New Zealand; 3 Centre for Biodiscovery and School of Biological Sciences, Victoria University of Wellington, Wellington, New Zealand; 4 Department of Pathology and Molecular Medicine, Otago University of Wellington, Wellington, New Zealand; 5 Coastal and Coast District Health Board, Department of Surgery, Wellington Hospital, Wellington, New Zealand

**Keywords:** Colorectal cancer, Prognosis, Biomarkers, Proteomics, Liver metastasis

## Abstract

Biomarkers are urgently required to support current histological staging to provide additional accuracy in stratifying colorectal cancer (CRC) patients according to risk of spread to properly assign adjuvant chemotherapy after surgery. Chemotherapy is given to patients with stage III to reduce the risk of recurrence but is controversial in stage II patients. Up to 25% of stage II patients will relapse within 5 years after tumor removal and when this occurs cure is seldom possible. The aim of this study was to identify protein biomarkers to stratify risk of spread of CRC patients. Laser micro-dissection was used to isolate cancer cells from primary colorectal tumors of stage II patients which did or did not metastasize within 5 years after surgical resection. Protein expression differences between two groups of tumors were profiled by 2D-DIGE with saturation CyDye labeling and identified using MALDI-TOF mass spectrometry. Evaluation of protein candidates was conducted using tissue micro array (TMA) immunohistochemistry on 125 colorectal tumor tissue samples of different stages. A total of 55 differentially expressed proteins were identified. Ten protein biomarkers were chosen based on p value and ratio between non metastasized and metastazised groups and evaluated on 125 tissues using TMA immunohistochemistry. Expression of HLAB, protein 14-3-3β, LTBP3, ADAMTS2, JAG2 and NME2 on tumour cells was significantly associated with clinical parameters related to tumour progression, invasion and metastasis. Kaplan–Meier survival curve showed strong expression of six proteins was associated with good CRC specific survival. Expression of HLAB, ADAMTS2, LTBP3, JAG2 and NME2 on tumour cells, was associated with tumour progression and invasion, metastasis and CRC specific survival may serve as potential biomarkers to stratify CRC patients into low and high risk of tumour metastasis. Combined methods of laser microdissection, 2D DIGE with saturation labelling and MALDI-TOF MS proved to be resourceful techniques capable of identifying protein biomarkers to predict risk of spread of CRC to liver.

## Introduction

Colorectal cancer (CRC) remains the leading cause of cancer deaths in the Western countries. The incidence and mortality of CRC are also rising in all developing countries [1, 2]. Metastatic dissemination from primary tumor accounts for over 90% of all colorectal cancer death [3]. Adjuvant chemotherapy has been shown to provide a significant improvement in patient survival, however this advantage is not available for all patients who could benefit from it due to inability of current standard method to accurately predict prognosis. Adjuvant chemotherapy for stage II CRC patients is still regarded as controversial [4–6]. About 25% of stage II CRC patients will develop metastasis after surgical removal of their primary tumor mainly to liver and 50–60% of stage III CRC patients will develop metastasis [7]. The overall survival rate for stage II CRC patients 5 years after surgery is approximately 70–80% and that for stage III patients is 30–60% [8].

Classic disease staging, which is currently the key prognostic indicator for CRC, involves examination of margin, depth of penetration of tumor through bowel wall and degree of lymph node involvement. Patient survival is strongly influenced by lymph node involvement. Recently histopathological features which include depth of tumor invasion in the wall of the colon, lymphovascular invasion (LVI), perineural invasion (PNI), poorly differentiated tumors, ≥ 13 lymph nodes sampled, bowel obstruction and localized perforation have been added to further define stage II CRC patients as high risk group [9].

Recovery and evaluation of lymph nodes in the resection specimen are, however, influenced by the method and quality of surgical resection, quality of pathologic evaluation, tumor related factors and patient factors [10, 11]. Variation in the assessment of lymph node status could lead to under-staging and as a result a falsely node-negative patient may not receive the potential benefit of adjuvant therapy. It is well recognized that staging by light microscopy alone is not sufficiently accurate to predict spread as significant variation with respect to clinical outcome exists within currently used stages.

Questions remaining to be answered include which patients will benefit from adjuvant chemotherapy and what chemotherapy to use to give most benefit for the patients. There is therefore urgent need for histological staging to be supported by molecular profiling of tumors to provide additional accuracy in stratifying patients for better disease management and ultimately improved survival.

Molecular profiling has been applied to explore prognostic signatures between patients [12, 13]. While results of some studies have been advanced to clinical use, others have failed to identify specific signatures, and have resulted in loss of confidence in the overall approach. In particular, tissue heterogeneity between patients contributes significantly to the “noise” and variability of samples. Non-cancerous cells within tumors such as fibroblasts, immune cells and blood cells have their own unique expression profile and differences in the proportions of these cells between tumor samples can strongly influence the overall molecular profile of the tumor, effectively masking any cancer cell-specific profile. Laser microdissection (LMD) allows populations of cells to be analyzed separately, minimizing contamination by other cell types, thereby reducing variation and allowing much more relevant and consistent comparison to be undertaken [14]. Two-dimensional differential gel electrophoresis (2D-DIGE) has been one of the approaches used in proteomic analysis [15–17]. The major advantages of DIGE are high sensitivity and linearity of the dyes utilized leading to significant reduction of inter-gel variability. The combined used of LMD and 2D-DIGE increases the potential for unambiguous identification of differentially expressed proteins while reducing biological variability and bias from inter gel variation and also minimizing contamination from non-tumor tissue. In addition, the use of a pooled internal standard increases quantification accuracy and statistical confidence [15, 16]. Saturation CyDye labeling of cysteine residues has been developed to improve the detection limits for proteins which are only approximately 5% of proteins present for the minimal labeling method. Saturation labeling has been shown to be at least 10 times more sensitive than minimal labeling, and in theory, should increase the potential for detecting low abundance proteins [17].

The aim of this study was to identify proteins differentially expressed between tumors from stage II CRC patients that tumor had metastasized or not metastasized within 5 years after removal of primary tumors. Selected candidates were then evaluated by TMA immunohistochemistry on tumor sections from patients with known clinical pathological parameters including survival times.

## Methods

### Surgical specimen tissue samples used for protein biomarker discovery

Primary colorectal tumors were collected from 11 sporadic stage II CRC patients whose tumors had not metastasized in 5 years after surgery (NM) and eight patients whose tumors had metastasized within 5 years post-surgery (M). All patients used in this study underwent surgical resection of their tumors at Wakefield Hospital, Wellington, New Zealand in 1998 to 2008. Tumor specimens were collected directly from theatre and immediately snap-frozen in liquid nitrogen and stored at -80^◦^C until used. Tissue collection and study was approved by the Central Regional Ethics Committee, Wellington in New Zealand, in accordance with the Helsinki Declaration of 1975.

Clinical samples and data sets used on this current study are not publicly available due to patient consent and Ethics but are available from the corresponding author on reasonable request.

### Data availability statement

Clinical samples and data sets used on this current study are not publicly available due to patient consent and Ethics but are available from the corresponding author on reasonable request.

### Laser microdissection

Tumour cells were isolated using LMD (Leica LMD6000, Leica Microsystem). Briefly, frozen tumour tissue was embedded in optimal cutting temperature (OCT) compound (Leica Microsystems, Wetzlar, Germany), cut into 20 µm thick sections using Leica CM1850 UV Cryostat (Leica Microsystems) at − 20 °C and mounted onto PET-membrane steel frame slides (Leica Microsystems). The sectioned tissues were fixed with 70% ethanol for 30 s, and stained in Harris’ haematoxylin (VWR, England) for 30 s then rinsed in water and air dried. All staining procedures were carried out on ice with all solutions supplemented with fresh 1 mM phenylmethanesulfonyl fluoride (Sigma, St Louis, MO, USA). The estimated total protein yield from one thousand cells was approximately one µg protein.

### Protein extraction and CyDye Fluor saturation dye labelling

Microdissected specimens were lysed in 100 µL lysis buffer (7 M urea, 2 M thiourea, 4% (w/v) [(3-cholamidopropyl) dimethylammonio]-1-propanesulfonate (CHAPS), 30 mM Tris (pH 8.5)). The lysates were centrifuged at 13,000 rpm for 10 min and the supernatants were collected. The proteins were purified using 2D-Cleanup kit (GE Healthcare, Uppsala, Sweden), and re-dissolved in 10 µL of the above lysis buffer for 2D-DIGE analysis. Protein concentrations were determined using Bradford microplate assay (Bio-Rad, Hercules, CA, USA).

Originally there were 11 NM and eight M tissue samples. Three of the 11 NM samples were too low in protein yield, and thus their resulting protein samples were pooled into one sample for 2D-DIGE analysis. Ultimately, nine protein samples from the NM patient group which consisted of eight individual samples and one pooled sample from three patients of low protein yield were used for 2D-DIGE analysis. Similarly four protein samples were obtained from the M patient group which consisted of three individual samples and one pooled sample from four patients for 2D-DIGE. One sample from the metastasized patient group was discarded because the protein concentration was too low.

CyDye DIGE Fluor saturation dye (GE Healthcare, Uppsala, Sweden) was used to label the proteins according to the manufacturer’s instructions. Briefly 5 μg protein extract was reduced with 2 nmol tris(2-carboxyethyl)phosphine for one h and then incubated with 4 nmol of CyDye for 30 min. The individual protein samples from microdissected tumour tissues (9 NM and 4 M) were labelled with Cy5. The internal standard, a pool composed of equal amount of proteins from all the samples (9 NM and 4 M), was labelled with Cy3 dye. All the labeling experiments were carried out in the dark at 37 °C.

### Two-dimensional differential gel electrophoresis

Five microgram of the internal standard labeled with Cy3 was added to 5 μg of each of the individual Cy5-labeled protein samples. This protein sample was then diluted with the rehydration buffer [7 M urea, 2 M thiourea, 4% w/v CHAPS, 1% (v/v) pharmalyte pH 3–11, 0.2% (w/v) dithiothreitol (DTT)] to a final volume of 200 μL. Eleven cm pH 3–11 non-linear Immobiline™ Drystrips (GE Healthcare) were placed in contact with the protein sample in the rehydration buffer overnight at room temperature in the dark. Isoelectric focusing (IEF, the first dimension of electrophoresis) was carried out using an Ettan IPGphor (GE Healthcare) as instructed by the manufacturer. Following IEF, the strips were equilibrated in equilibrium buffer (100 mM Tris pH 8.0, 6 M urea, 2% SDS, 30% glycerol) containing 0.5% DTT, and subsequently 4.5% iodoacetamide, for 10 min each. The equilibrated strips were electrophoresed on standard continuous SDS-PAGE gels (Criterion™XT 4–12% Bis Tris precast gel, Bio Rad) at 200 V for 50 min. The SDS PAGE gels were scanned using a fluorescent Fujifilm FLA-5100 scanner (Fujifilm, Tokyo, Japan) with 50 μ and 12 bit resolution. A 532 nm laser and a PBG/570DF20 emission filter were used to scan the Cy3 images and a 635 laser and a DBR1/R665 emission filter were used to scan the Cy5 images.

Delta 2D software (version 3.6, DECODON GmbH, Greifswald, Germany) was used to analyze the 2D-DIGE images. Gel images were warped using the In-Gel Standard Warping Strategy with all Cy3 internal standard gel images warped to the master gel image using the EXACT mode. Gel images were fused and spot detection was carried out on the fused gel image to produce a consensus spot pattern. All spot quantities were normalized to the internal standard resulting in quantities described by the formula %V (Spot X of sample A) = rel V (Spot X of sample A)/rel V (Spot X of internal standard), where rel V is the absolute volume of the spot divided by the cumulative absolute volume of all spots on the same image. Differentially expressed proteins were determined by Student’s *t* test based on log2 transformed protein spot quantities with *p* value ≤ 0.05 and the ratio of the protein spots’ quantities of a matched protein in NM over M fold change ≥ 1.5 or ≤ 0.67.

### Protein identification

Briefly, 400 μg of the pooled proteins from all samples were separated on a 2D gel as above but stained with Coomassie brilliant blue solution (0.05% colloidal CBB G-250, 17% ammonium sulfate, 2% orthophosphoric acid, 34% methanol) overnight and scanned using a Molecular Dynamics Scanner, Personal Densitometer SI (Sunnyvale, CA, USA) with 50 µ and 12 bit resolution. Proteins spots of interest were excised using a 1.5 mm diameter One Touch Plus Spot Picker (The Gel Company, San Francisco, CA, USA). In-gel digestion of protein spots was performed as described previously [18]. Briefly, the gel spots were placed in a 96-well plate and detained in 0.1 mL of destaining solution (50 nM ammonium bicarbonate, 50% methanol) overnight. Gel plugs were washed three times in the destaining solution for 20 min each and finally washed with acetonitrile. Spots were air dried in a SpeedVac (for 60 min and then digested in 5 μL 20 mM ammonium bicarbonate with 0.1 μg sequencing grade bovine trypsin (Roche Diagnostic, Manneheim, Germany) for five h at RT. Protein digests were extracted with 20 μL of 0.1% trifluoroacetic acid in 50% acetonitrile twice and subsequently with 100% acetonitrile for 20 min each. The combined 60 μL extracts were transferred to a new 96-well plate and air dried for at least 14 h. Dried peptides were reconstituted in 1 μL of saturated α-cyano-4-hydroxycinnamic acid (Sigma-Aldrich) solution containing 0.1% trifluoroacetic acid, 50% acetonitrile and 0.05% Calibration mixture 2 (Sequazyme™ Peptide Mass Standard Kit, Applied Biosystems, CA, USA) and deposited on the surface of a matrix-assisted laser desorption ionization time of flight mass spectrometry (MALDI-TOF MS) plate (Applied Biosystems). MALDI-TOF MS was performed using a Voyager-DE PRO Biospectometry™ Workstation (Applied Biosystems) as described previously [19]. Spectra were acquired using positive reflector mode with the following voltage setting: accelerating 20,000 V; grid, 75%; guide wire, 0.005; delay time, 180 ns. Each spectrum consisted of 110 laser shots and mass range from 800 to 3500 Da. Spectra were processed using Data Explorer™ (Applied Biosystems). Three peaks from Calibration Mixture 2 of Sequazyme™ Peptide Mass Standards (1296.6853, 2093.0867 and 2465.1989) were used for internal calibration. Following peak de-isotoping, a peak list of monoisotopic peptide masses was generated using a peak detection threshold ranging from two to 10% of maximum peak area. The resulting monoisotopic peptide masses were searched against the UniProtKB human protein database as of 1 November 2009 using Aldente (http://kr.ezpasy.org/tools/aldente/) with the following searching parameters: maximum missed tryptic cleavage 1, static carboxymidomethylation modification of cysteine (+ 57.02 Da) and dynamic oxidation of methionine (+ 15.99 Da), peptide mass tolerance 0.2 Da.

### Tissue microarray and immunohistochemical stain

Initially, five tissue microarray (TMA) blocks containing a total of 126 CRC cases were constructed. Each TMA consisted of up to 26 tissue cores with a single tissue core per patient tumour. Before constructing TMA, a four µm thick section was obtained from each tumour block, stained with haematoxylin and eosin and examined by a pathologist (Rose Miller). Areas of sampling (AOS) were defined and marked on the microscope slide by the pathologist and used to guide the location of tissue of interest for core punching. TMA was constructed using the Beecher Automated Tissue Arrayer (ATA-27, Beecher Instruments, USA). A tissue core with a diameter of one mm was punched from the donor tissue block under guidance of the reference slides and transferred to a recipient paraffin block (array margin of 10 × 20 mm).

Four µm thick tissue sections were cut from each TMA block. Tissue sections were serially rehydrated. Antigen retrieval was performed by submerging the slides in sodium citrate buffer (10 mM sodium citrate, 0.05% Tween 20, pH 6.8) in a pressure cooker for 10 min at boiling point. After cooling to room temperature, tissue sections were incubated in 3% H_2_O_2_ in Phosphate Buffer Saline-Tween-20 (PBS-T) for 10 min to block endogenous peroxidase. Staining was performed using R.T.U Vectastain^®^ Universal Quick kit (Vector Laboratories, Inc, Burlingame, CA) as follows. Briefly sections were sequentially blocked in 2.5% normal horse serum for 10 min, incubated in primary antibody diluted in Phosphate Buffer Saline (PBS) for 1 h, washed in PBS-T for five min, biotinylated in pan-specific universal secondary antibody for 10 min, washed in PBS, incubated in streptavidin/peroxidase complex for 5 min, washed in PBS and incubated in the DAB substrate kit. Primary antibodies for candidate proteins (Table 2) were purchased from Santa Cruz 14-3-3β (A-6) (sc-25276) at 1:40; nm23-H2 (L-14) (sc-14789) 1:150; MRCL3/MRCL2 (A-20) (sc-9449) at 1:50; SRPX (N-18) (sc-10700) at 1:100; Wnt-5a (C-16) (sc-23698) at 1:50; Jagged2 (H-143) (sc-5604) at 1:100; ADAMTS-2 (18Q) (sc-100479) at 1:200; MHC class I (F-3) (sc-55582) at 1:600; LTBP-3 (H-21) (sc-98276) at 1:250 and RECK (28) (sc-136270) at 1:30. Slides were counterstained by dipping in hematoxylin three times, rinsed with water, dehydrated in xylene and finally mounted with DPX- mounting media (IHC World, LCC).

### Image analysis and scoring

Expression of 10 proteins (Table 1) in this study was graded and scored blind by two pathologists (Rose Miller and Corinne Glenn). The intensity of proteins was scored from 1 to 3 with one for weak or none, two for moderate and three for strong and correlated with clinical parameters [20].

**Table 1:** Differentially expressed proteins of interest detected by 2D-DIGE that were chosen for evaluation by immunohistochemistry

No.	Gene name	Uniprot accession number	Protein name	Fold change (NM/M)*	*p* value (NM/M)	Peptides matched	Protein sequence coverage (%)
1	LTBP3	Q9NS15	Latent-transforming growth factor beta binding protein 3	1.7	0.05	19	16
2	HLAB	P30475	HLA class 1 histocompatibility antigen, B39 alpha chain	2.5	0.03	8	21
3	ADAMTS2	O95450	A disintegrin and metalloproteinase with thrombospodin motifs 2	1.5	0.01	14	22
4	RECK	O95980	Reversion-inducing cysteine-rich protein	1.8	0.03	15	19
5	WNT5A	P41221	Wnt-5a protein	0.56	0.06	9	22
6	14-3-3-β	P31946	14-3-3 protein beta/alpha	0.67	0.05	9	37
7	SRPX	P78539	Sushi repeat-containing protein SRPX	1.5	0.02	14	26
8	NME2	P22392	Nucleoside diphosphate kinase B (nm23H2)	1.6	0.005	8	38
9	MRLC 3	P19105	Myosin regulatory light chain MRLC3	0.67	0.025	7	46
10	JAG2	Q9Y219	Protein Jagged-2	1.7	0.05	23	20

Note: Student’s *t* test based on log2 transformed spot volumes between non-metastasized (NM) and metastasized (M) tumors

### Statistical analysis

Student’s *t* test was used to assess differential expressions of proteins in tumor cells between non-metastasized and metastasized groups. Statistical analyses on the expressions of proteins in tumor tissues were performed using SPSS (version 17). The association between protein immunoreactivities and patient clinicopathological parameters were assessed by χ^2^ test. The impact of individual protein on patient survival was examined by Kaplan–Meier curve analysis and the statistical significances were determined using log-rank test. A *p* value ≤ 0.05 was considered significant.

## Results

### Identification of differentially expressed proteins by 2D-DIGE

**Fig. 1:**
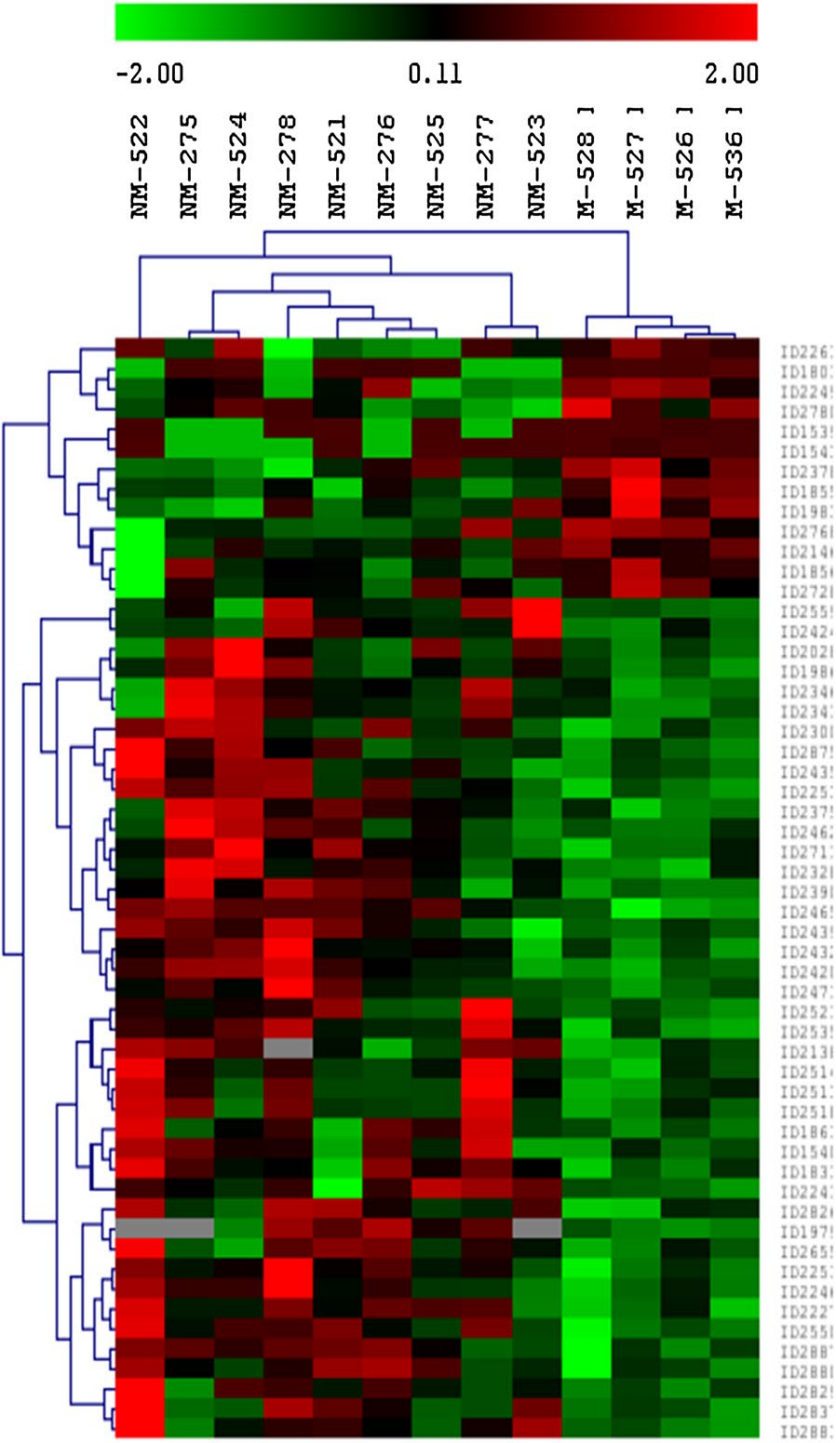
Hierarchical clustering dendrogram and heat map of differentially expressed proteins (p ≤ 0.05; fold change ≥ 1.5 or ≤ −1.5*) between the non-metastasized (NM) and metastasized (M) patient groups of colorectal cancer within 5 years after the primary tumors were removed. The dendrogram and heat map illustrate the protein expression patterns between the two groups with the segregation and alignment of proteins with similar functional groups and clustering of experimental groups with similar disease pathology. Red indicates a high relative level of protein expression, black for median value and green indicates low relative level of protein expression. *The fold change is defined as the ratio of the protein spots’ quantities of a matched protein in NM over M if its value was greater than 1, or the negative reciprocal of the ratio if its value was smaller than 1

A total of 1123 protein spots were detected and matched on the analytical gels between the two groups by 2D-DIGE. Out of 1123 protein spots, 55 showed significantly different volume changes between the non-metastasized and metastasized groups [*p* ≤ 0.05, fold change (NM/M) ≥ 1.5 or ≤ −1.5]. Figure 1 shows the unsupervised hierarchical clustering analysis of the two groups based on the volumes of differentially abundant protein spots. The protein expression profiles in the metastasized group showed some consistent changes among individuals. The protein expression profiles in the non-metastasized group and metastasized group showed to be heterogeneous among its individual. Protein expression patterns between two groups of sample were distinct.

### Evaluation of differentially expressed proteins by immunohistochemistry

Ten differentially expressed proteins of interest detected by 2D-DIGE were chosen based on low *p* value (*p* ≤ 0.05) and high fold change between the non-metastasized (NM) and metastasized (M) patient groups of colorectal cancer within 5 years after the primary tumors were removed and also their known roles in cancer progression and metastasis and further evaluated using TMA immunohistochemistry of 125 colorectal tumor tissue samples. A summary of these proteins is presented in Table 1.

**Fig. 2:**
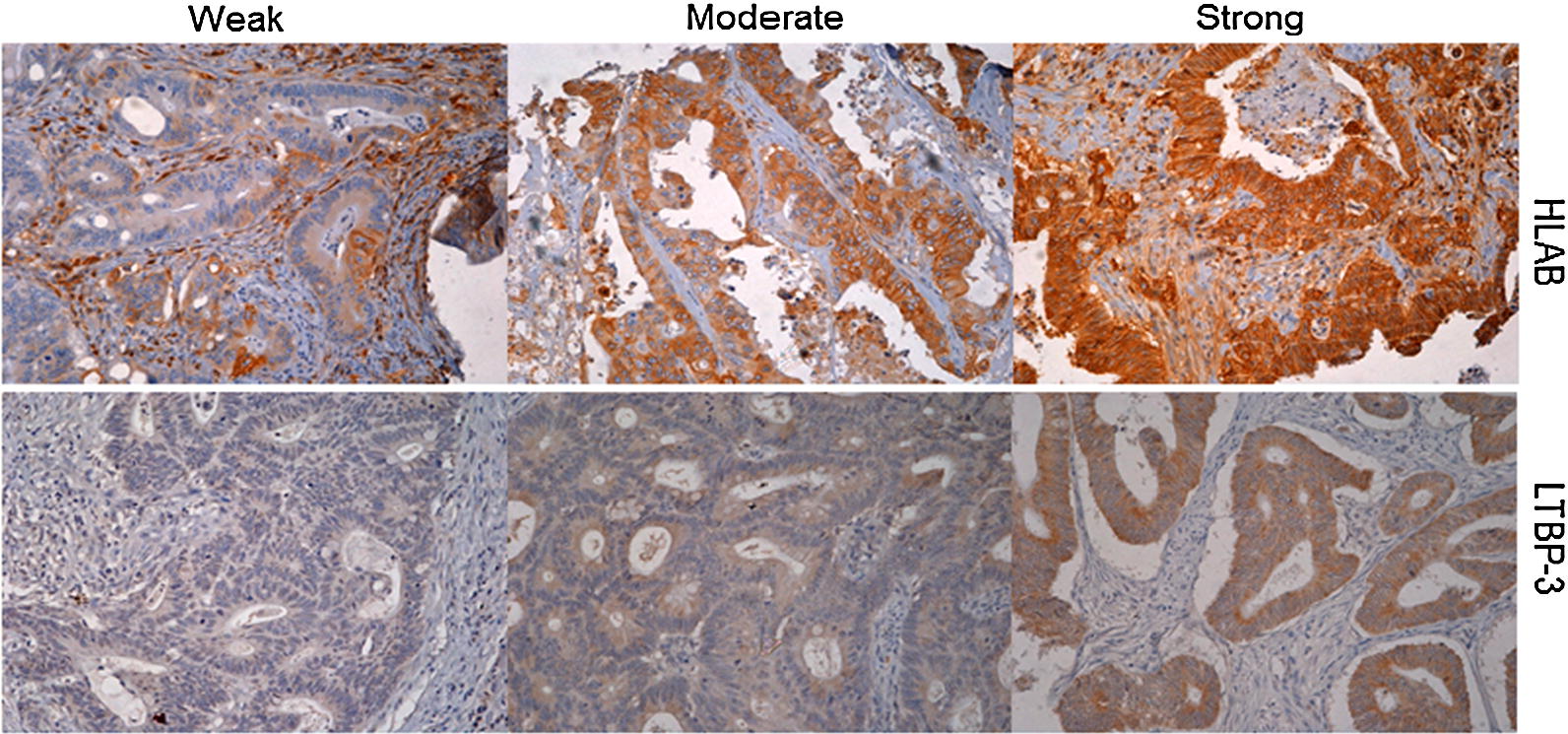
Representative CRC tissues showing low, moderate and strong staining pattern of HLAB (top) and LTBP3 (bottom) proteins on tumour cells by immunohistochemistry (DAB substrate brown) (×20 objective). Sections were counterstained with hematoxylin (blue)

The expression (weak, moderate and strong) of two representative proteins, HLAB and LTBP3 on CRC tissue using immunohistochemistry is illustrated in Fig. 2.

The association between clinical pathological parameters with the expression levels of ten proteins in tumor tissues of 125 TMA immunohistochemistry is shown in Table 2. Expression of six proteins, HLAB, 14-3-3β, ADAMTS2, LTBP3, NME2 and JAG2 on colorectal tumour cells significantly correlated with some clinical pathologic factors, whileWNT5A, MRLC3, RECK and SRPX showed no association.

**Table 2:** Correlations of protein expression levels with clinicopathological features

Clinico-path parameters	No of cases (%)	HLAB	14-3-3 β	LTBP-3	ADAMTS2	Wnt-5a	MRLC 2/3	Jagged-2	nm23 H2	SRPX	RECK
Age		0.923	0.273	0.132	0.246	0.437	0.173	0.644	0.745	0.145	0.559
< 65	35 (29)										
≥ 65	84 (71)										
Gender		0.556	0.363	0.178	0.938	0.562	0.709	0.515	0.866	0.681	0.215
F	59 (50)										
M	60 (50)										
Tumor location		0.230	0.318	0.231	0.444	0.317	0.078	0.589	0.736	0.534	0.036
Colon	82 (69)										
Rectum	37 (31)										
Histological type		0.106	0.001*	0.952	0.621	0.192	0.792	0.044*	0.158	0.526	0.659
Non mucinous	102 (86)										
Mucinuo	16 (14)										
Histological grade		0.020*	0.031*	0.743	0.326	0.266	0.402	0.646	0.032*	0.149	0.327
High grade	95 (81)										
Low grade	23 (191)										
Vascular invasion		0.556	0.200	0.500	0.730	0.279	0.584	0.038*	0.048*	0.401	0.166
Negative	90 (76)										
Positive	29 (24)										
Perineural invasion		0.017*	0.466	0.194	0.457	0.300	0.598	0.664	0.080	0.925	0.747
Negative	109 (92)										
Positive	9 (8)										
TNM stages		0.394	0.226	0.647	0.664	0.508	0.403	0.374	0.147	0.184	0.257
I	20 (17)										
II	42 (36)										
III	34 (29)										
IV	21 (18)										
Distant metastasis		0.307	0.850	0.003*	0.013*	0.503	0.833	0.808	0.418	0.681	0.210
No	89 (75)										
Yes	29 (25)										
Nodal status		0.227	0.417	0.403	0.336	0.894	0.101	0.283	0.413	0.463	0.194
Negative	66 (58)										
Positive	47 (42)										
Depth of invasion		0.426	0.041*	0.497	0.855	0.095	0.152	0.054	0.092	0.110	0.245
T1/T2	23 (20)										
T3/T4	93 (80)										
5-year recurrence^#^		0.104	0.120	0.075	0.041*	0.671	0.781	0.763	0.761	0.673	0.654
Recurrence free	58 (79)										
Recurrence	15 (21)										
Cause of death		0.025*	0.139	0.005*	0.055	0.380	0.650	0.488	0.144	0.390	0.265
CRC	37 (30)										
Other cause	87 (70)										

*p < 0.05 is considered significant

# Presence of absence of local or distant metachronous recurrence within 5 year follow-up

HLAB was significantly associated with histological grade (*p* = 0.020); PNI (*p* = 0.017) and cause of CRC death (*p* = 0.025). Expression of 14-3-3 β protein significantly correlated with mucinous (*p* = 0.001), histological grade (*p* = 0.031) and depth of tumour penetration (*p* = 0.041). LTBP3 expression on tumour cells was significantly associated with distant metastasis and CRC cause of death (*p* = 0.003 and p = 0.005 respectively). ADAMTS2 was significantly correlated with distant metastasis (*p* = 0.013), 5 year disease recurrence (*p* = 0.041) and at border line for CRC specific cause of death (*p* = 0.055). JAG2 expression on tumour cells was significantly associated with mucinous (*p* = 0.044) and LVI (*p* = 0.038) and NME2 were significantly correlated with histological grade and LVI at *p* = 0.032 and *p* = 0.048 respectively.

**Fig. 3:**
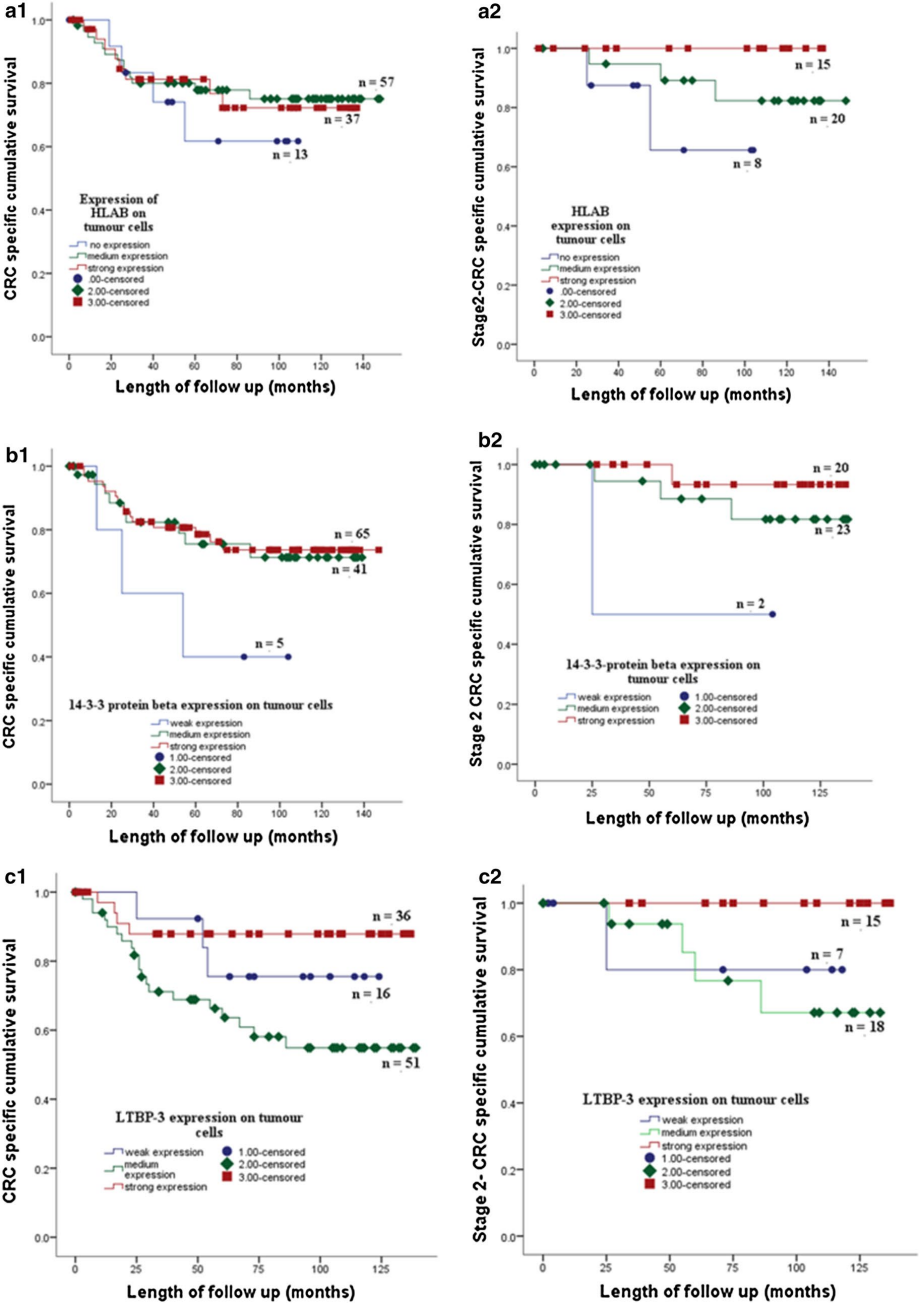
The expression of HLAB (**A1**) 14-3-3 protein beta (**B1**) and LTBP3 (**C1**) of the tumour cells of stage 1, 2, 3 CRC patients in a cohort of 125 tumor tissues is associated with CRC specific survival. Strong expression of HLAB, 14-3-3 protein beta and LTBP3 on cancer cells of stage 2 CRC patients showed better CRC specific survival than moderate and or weak expression of the proteins respectively (**A2**, **B2**, **C2**)

**Fig. 4:**
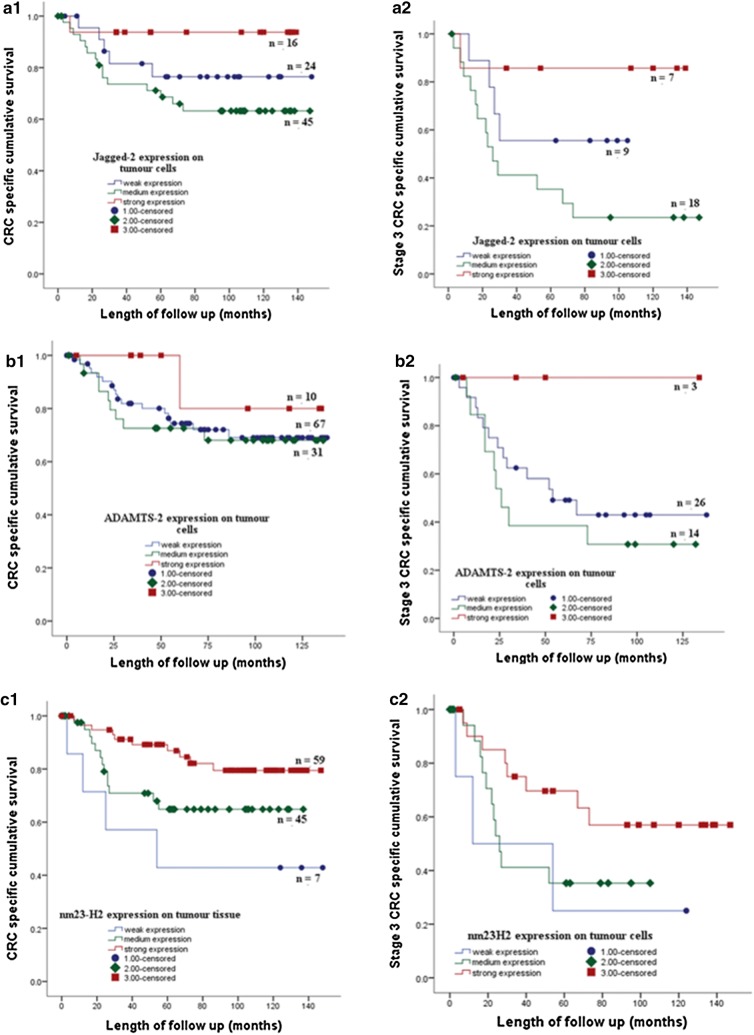
The expression of JAG2 (**A1**) ADAMTS2 (**B1**) and NME2 (**C1**) of the tumour cells of stage 1, 2, 3 CRC patients in a cohort of 125 tumor tissues is associated with CRC specific survival. Strong expression of JAG2, ADAMTS2 and NME2 on cancer cells of stage 3 CRC patients showed better CRC specific survival than moderate and weak expression of the proteins respectively (**A2**, **B2**, **C2**)

The association of protein expression with cancer specific survival was assessed by univariate analysis using Kaplan–Meier curve and log-rank test. Kaplan Meier survival curve showed the expression of HLAB, 14-3-3β, ADAMTS2, LTBP3, NME2 and JAG2 on colorectal tumour cells associated with CRC specific survival. Strong expression of the proteins was associated with good CRC specific survival. The association between expression of the proteins and CRC specific survival also shown on CRC patients with the stage 2 and 3 (Figs. 3 and 4).

## Discussion

CRC remains a major public health burden worldwide. Australia and New Zealand have the highest incidence rate of CRC, which account the third most common diagnosed cancer and the second cause of cancer death [21]. The incidence of CRC is also rising in the developing countries [1, 2]. About 15–30% of patients with stage II and 50–60% of patients with stage III will eventually develop distant metastases after their primary tumor is removed which then results in poor outcomes and subsequent death [22]. Adjuvant chemotherapy, however, is only common practice for stage III patients and still debated for stage II patients [4–6]. Classical histological staging remains the gold standard practice to measure CRC prognosis. The American Society of Clinical Oncology [7] has added histopathological features including depth of tumor infiltration, < 13 lymph nodes sampled, perineural invasion, lymphovascular involvement and poorly differentiated histology tumor to further subgroup stage II patients into low and high risk category and therefore adjuvant chemotherapy administration is considered. However, the benefit of adjuvant chemotherapy for stage II patients in improving 5 year survival was only about 5%. Additional molecular markers are urgently required to further identify high risk patients so that adjuvant chemotherapy will have benefit to the patients.

In this study we have combined LMD and 2D-DIGE application to profile proteins from tumor cells of stage II CRC patients whose tumors did or did not metastasize within 5 years of first surgical intervention. Tissue heterogeneity between patients and presence of non-cancerous cells within tumors contributes significantly to the “noise” and variability of samples thereby strongly affect molecular profile of tumour cells. Laser microdissection allows populations of cells to be analyzed separately to minimize contamination from other cell types, thereby reducing variation and allowing more relevant and consistent comparison [14].

The 2D-DIGE approach is used to minimize variations between gels and improve reproducibility which can be problematic in traditional 2-DE. Increased confidence in detection of proteins was facilitated by running an internal standard in every gel. The strategy of saturation dye labeling is superior to minimal label dye approach where limited quantities of protein sample are available [17]. Due to limited protein yield available from LMD samples we used saturation dye labeling and 2D-DIGE for this program. Saturation labeling has been shown to be more sensitive than minimal labeling in protein detection, which in theory should increase the potential for detecting low abundance protein [23]. Protein profiles from metastasized samples were more homogenous than those of non-metastasized samples. Proteins with differential expression patterns between metastasized and non-metastasized tumor were markedly observed. Six of the 10 (60%) proteins examined using TMA immunohistochemistry, HLAB, protein 14-3-3β, LTBP3, ADAMTS2, JAG2 and NME2, were significantly associated with clinical parameters which have shown to relate with tumor progression, invasion and metastasis. Four proteins, WNT5A, MRLC3, RECK and SRPX did not have association with clinical parameters used in this study. Using combination of LMD and saturation dye labeling and 2D-DIGE, we identified proteins which have been reported to associate with cancer using more advanced as well as 2-DE proteomic techniques. Protein 14-3-3 β was found by Zang et al. [24] using more advanced proteomic techniques while NME2 was identified differentially expressed in lung adenocarcinoma and bone metastasis lung cancer using 2-DE and MALDI-TOF-MS [25]. In addition, we also identified ADAMTS2 and LTBP3. These proteins have been reported to associate with cancer progression, however, the expression has not been previously reported in clinical samples.

HLAB belongs to major histocompatibility complex proteins (MHC) class I. MHCs are cell surface glycoproteins that play a fundamental role in the regulation of immune responses. It has been widely known that the availability of MHC class I on tumor cell membrane is important for enabling CD8+ cytotoxic T-lymphocytes recognition and binding to tumor antigen peptides [26, 27]. The loss of MHC class I results in escape from recognition and destruction by CTLs. CD8+ cells play crucial roles in anti-tumor responses. Down-regulation of HLA class I in cancers has been reported to associate with poor prognosis for example in rectal cancer [28] and ovarian cancer [29]. Our study showed that colorectal tumor which did not metastasize had stronger expression of HLAB than tumor which metastasis. Loss or low expression of HLAB in CRC tissue was significantly associated with distant metastasis, disease recurrence and CRC specific survival. In addition, Kaplan Meier survival analysis showed that no expression of HLAB on tumour cells showed worst prognosis.

ADAMTS (A disintegrin and metalloproteinase with thrombospondin motifs) enzymes are secreted multi-domain matrix-associated zinc metalloendopeptidases. There are 19 ADAMTs. ADAMTS2, 3 and 14 are procollagen N-endopeptidase enzymes. They play crucial roles in collagen type I and III fibrils maturation and formation [30]. We found that the expression of ADAMTS2 was down-regulated in the tumour cells of patients that metastasized compared to those that did not metastasize. Expression of ADAMTS2 was significantly associated with distant metastasis and at borderline value with cause of colorectal cancer specific death. Using Kaplan-Meier survival curve and log-rank analysis showed that patients whose tumor loss of ADAMTS2 showed significantly shorter cancer survival than those whose tumors expressed ADAMTS2. ADAMTS2 was reported to have anti proliferation and anti angiogenic activity and induced apoptosis independent of its catalytic activity [31]. In addition, ADAMTS1 was described as having tumor-protective effects and angiogenesis inhibitory enzyme due to its capacity to restrain endothelial cell proliferation [32]. We reported for the first time that the expression of ADAMTS2 in clinical samples, CRC tissue and blood, significantly associated with liver metastasis and good prognosis. Using both cell culture and a mouse model of vascularization of tumours, Dubail et al. [31] showed that ADAMTS2 inhibited proliferation and angiogenesis of endothelial cells and that the growth of tumor with ADAMTS2 treatment had less vascularization compared to control animals.

Ligand-induced Notch signaling has been implicated in cancer biology. JAG2 is one of the ligands in Notch signaling. In mammalian cells, Notch signaling consists of four receptors (Notch1–Notch4), three delta like ligands (DLL1, DLL3 and DLL4) and two serrate-like ligands (JAG1 and JAG2) [33].

Although it has been reported that up regulation of JAG2 expression was associated with the progression of tumour including in pancreatic [34], lung [35] and bladder cancer [36], the role of JAG2 in cancer remains unclear [33, 36]. In contrast, our findings showed that expression of JAG2 on tumour cells significantly associated with histological type of tumour, and vascular invasion. Strong expression of JAG2 in tumour cells correlated with good CRC specific survival.

14-3-3 proteins are highly conserved protein family. There are seven family members of the proteins in mammals, -β,-γ, -ε, -σ, - ζ, -τ and –η. 14-3-3 proteins bind a variety of functionally diverse signaling proteins including phosphatases, trans-membrane receptors and kinases which contribute to survival and apoptotic signaling, cell growth, tumour suppression and cancer development [37]. Loss of 14-3-3σ has been reported to have tumour-suppressing activities in breast cancer [38]. In contrast 14-3-3-ζ has been suggested to contribute to tumour invasion and migration [37]. Our study found that the expression 14-3-3-β protein on tumour cells significantly associated with histological type and grade of CRC tumour and depth of invasion of tumour cells. Stronger expression of 14-3-3-β protein associated with better CRC specific survival.

NM23 family proteins are highly conserved proteins. There were eight genes in the human genome encoding NM23 family proteins and suggested as suppressor of tumour metastasis [39]. NME2 protein has anti tumorigenic activity thru blocking ERK pathway [40]. Our study showed that the expression of NME2 in tumour cells significantly associated with histological grade of tumour and vascular invasion. Strong expression of NME2 in tumour cells indicated good CRC specific survival.

LTBPs are important component of extracellular matrix (ECM) that interact with fibrillin microfibrils. There are four LTBPs isoform in human (LTBP1, -2, -3 and -4) [41]. Our findings identified LTBP3 in tumour cells. LTBP-3 on tumour cells of stage II patients whose tumour did not metastasize and those whose tumour metastasized to the liver were differentially expressed. The expression of LTBP3 in the tumour cells significantly associated with distant metastasis and CRC cause of death. Strong expression of LTPB3 in tumour cells showed good CRC survival. LTBP3 was reported [41] to promote early metastasis during cancer cell dissemination by inducing intravasation supporting angiogenic vasculature within developing primary tumours. Report on LTBP3 in clinical samples has not been reported elsewhere. The role of LTBP3 in cancer is understudied.

## Conclusions

Combined methods of laser microdissection, 2D DIGE with saturation labelling and MALDI TOF/TOF proved to be resourceful methods capable to show proteins differences of stage II CRC patients whose tumours did not metastasis with those whose tumours metastasized to liver. Proteins, HLAB, ADAMTS2, LTBP3, JAG2 and NME2 were significantly associated with tumor progression, vascular invasion and distant liver metastasis. These proteins are potential biomarkers to current classic pathological staging for separating patients with low and high risk of tumour spread. Individual or combined proteins may potentially serve as additional marker to current classic staging to identify distant liver metastasis and provide be better therapeutic CRC management to reduce cancer spread and decrease mortality of colorectal cancer.
